# Comparison of Kinematic Alignment and Mechanical Alignment in Total Knee Arthroplasty: A Meta‐analysis of Randomized Controlled Clinical Trials

**DOI:** 10.1111/os.12826

**Published:** 2020-10-25

**Authors:** Zhi‐xiang Gao, Neng‐ji Long, Shao‐yun Zhang, Wei Yu, Yi‐xin Dai, Cong Xiao

**Affiliations:** ^1^ Department of Orthopaedics The Third Hospital of Mianyang, Sichuan Mental Health Center Mianyang China

**Keywords:** Alignment, Knee joint, Meta‐analysis, Total knee arthroplasty

## Abstract

The aim of this study was to estimate whether kinematic alignment (KA) improves knee function or clinical outcomes compared with mechanical alignment (MA) in the short term after total knee arthroplasty (TKA). We searched the literature for randomized controlled trials published before January 2020 from PubMed, EMBASE, Google, Web of Science, Cochrane Library, and other databases. The observation markers included “The Western Ontario and McMaster Universities (WOMAC) Osteoarthritis Index,” “Knee Society Score (KSS),” “Oxford Knee Score (OKS),” “combined Knee Society Score (KSS),” “Knee injury and Osteoarthritis Outcome Score (KOOS),” “European Quality of Life Measure‐5 Domain‐5‐Level (EQ‐5D‐5L),” range of motion (ROM), lower limb alignment, ligament release, and complications. A total of 11 randomized controlled trial studies were included in the study. During the follow‐up of 6–24 months, the KA‐TKA group was superior to the MA‐TKA group in terms of WOMAC scores, combined KSS, KSS, knee function scores, and knee range of flexion, but there was no significant difference in EQ‐5D‐5L, KOOS, KOOS (symptoms, pain, ADL, sports, and quality of life), complications, knee range of extension, hip‐knee‐ankle (HKA) angle, tibial component slope angle, lateral distal femoral angle (LDFA) or medial proximal tibial angle (MPTA) angle between the MA‐TKA group and the MA‐TKA group (*P >* 0.05). Our meta‐analysis revealed that the incidence of ligament release in the MA‐TKA group was higher than that in the KA‐TKA group. This meta‐analysis shows that the KA‐TKA group had better clinical outcomes and knee range of flexion than the MA‐TKA group at short‐term follow‐up.

## Graphical abstract

Although the survival rate of MA‐TKA has improved, approximately 20%–25% of patients remain unsatisfied with the outcome.Therefore, this article to evaluate whether the clinical outcome of KA‐TKA is better than that of MA‐TKA.

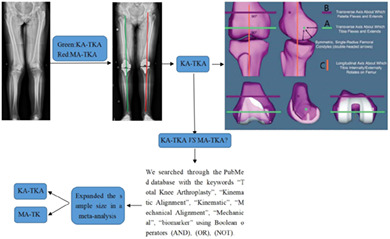

## Introduction

Osteoarthritis (OA) is the most widespread joint disease in the elderly, and knee osteoarthritis (KOA) is more frequent than OA of the hip or ankle^1^, ^2^. It has been predicted that in 2020, OA will be the fourth most common cause of disability worldwide^3^. At present, the first choice for severe joint diseases (Kellgren–Lawrence score ≥ 3) is total knee arthroplasty (TKA), which can relieve joint pain, correct deformity, and improve joint function, and many studies have suggested that the long‐term survival rate could reach more than 90% after 15 years^4^, ^5^, ^6^. It has been estimated that by 2030, every year, 3.8 mn people will undergo TKA^7^. Although the survival rate of TKA has improved, approximately 20%–25% of patients remain unsatisfied with the outcome^8^.

**Fig. 1:**
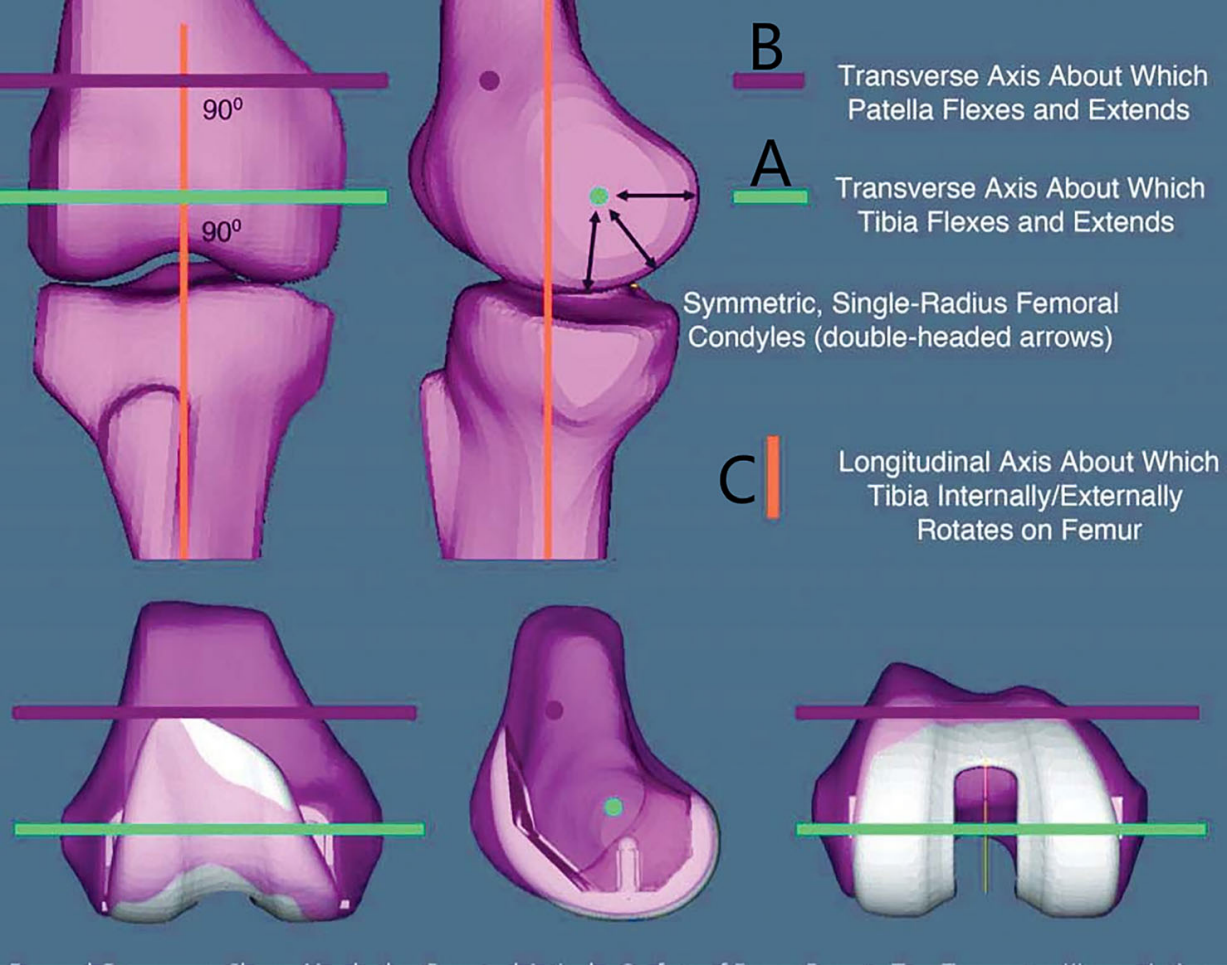
The kinetic characteristics of normal knee are governed by three axes. (Photo credit: Dossett HG, Swartz GJ, Estrada NA, *et al*.^13^

Traditional mechanical alignment (MA) has been used in TKA for more than 30 years, and it is still common worldwide. It is generally believed that a hip–knee–ankle (HKA) angle within less than 3° of the neutral mechanical axis is essential for postoperative limb recovery after TKA^9^, ^10^. With the development of knee biomechanics, however, many people have assumed that MA does not entirely restore normal lower limb alignment, may alter the normal kinematics of knee motion and so contribute to some of the most serious ramifications. Some foreign scholars^11^, ^12^, ^13^ have found that the kinetic characteristics of the normal knee are governed by three axes (Fig. 1). One is the transverse axis of the femur; during knee flexion and extension, the tibia moves around the transverse axis of the centerline of the medial and lateral condyle of the femur^14^, ^15^, ^16^. Another is the patellar transverse axis, a transverse axis around which the patella rotates during knee flexion and extension; its spatial position is anterior and proximal of the central transverse axis of the femur^17^, ^18^. The last axis is the longitudinal axis of the tibia, which is perpendicular to the transverse axis of the femur; the tibia rotates internally and externally around the longitudinal axis of the tibia^16^, ^17^, ^18^. Therefore, the overall mechanical alignment takes into account the two‐dimensional alignment of the parts with the center of the femoral head, knee, and ankle. Kinematic alignment (KA) is different from mechanical alignment (MA) in that it mainly considers the three‐dimensional alignment of the components relative to the knee and involving movement in 6° of freedom (6‐DOF: front‐to‐back, proximal‐to‐distal, internally‐to‐externally, extension‐to‐flexion, varus‐to‐valgus, internal‐to‐external rotation)^12^, ^13^. Based on this theory, in 2006, Howell *et al*.^19^ proposed kinematic alignment in TKA (KA‐TKA). The primary purpose of KA‐TKA is to control the kinematics of the patella and tibia relative to the femur by restoring the above mentioned three axes of the distal femur and the proximal tibia rather than merely generating a neutral HKA angle.^12^ Meanwhile, Howell *et al*. ^12^ also proposed an osteotomy and guide apparatus customized for KA‐TKA patients. However, this technique requires a highly reliable method. In other words, pre‐operative three‐dimensional scans of the articular surface of the femoral and tibia by MRI, in an MRI scan, require flexion‐extension axis (FEA) of tibia vertically to the sagittal. In addition to this, manual instrument techniques were also reported to be effective^20^. But, the following important aspects should be carefully evaluated: line of force of the lower limbs; anatomic axis of the knee; internal‐external rotation of the tibia component relative to the femur; valgus or varus degrees placement of the tibia component; anatomic axis of the knee^21^. Howell *et al*.^22^ tested two methods for this purpose, and found that the accuracy was similar with and without Patient Specific Cutting Blocks. Some studies^19^, ^23^, ^24^, ^25^, ^26^, ^27^ have shown that KA‐TKA is more likely to restore normal knee kinematics and that its clinical outcome is quite favorable compared with MA‐TKA. And they consistently concluded that KA‐TKA could significantly improve patient's quality of life, higher mean flexion range angle, and reduced the prevalence of pain, joint stiffness, and instability^28^. However, KA‐TKA has some potential problems: an increased risk of patellar instability and polyethylene wear^29^, ^30^.

While relevant meta‐analyses have been published in recent years, these studies included randomized controlled trials, case reports, and systematic reviews^27^, ^31^. Two systematic reviewss^27^, ^31^ have analyzed the kinematic and mechanical alignment techniques in TKA. These two meta‐analysess^27^, ^31^ included retrospective observational studies and randomized controlled trials (RCTs). Both authors agree that the KA‐TKA provided better functional outcomes in addressing pain and improving function. Waterson *et al*.^32^ have also analyzed 71 KOA patients undergoing TKA in which 36 patients underwent kinematic alignment treatment and 35 patients received mechanical alignment. The results showed that the two groups had similar function 1year post‐operatively. Another recent randomized controlled trial showed that the KA‐TKA offered better pain relief and higher mean flexion range angle than the MA‐TKA at two years^33^. As the quality of data in these studies is often limited, differences in results exist. It is still uncertain whether the benefits of KA‐TKA are superior to those of MA‐TKA. Therefore, we systemically analyzed the available data after searching the literature for randomized controlled trials to evaluate whether the clinical outcome of KA‐TKA is better than that of MA‐TKA.

## Materials and Methods

### *Search Strategy*

This meta‐analysis method was based on the Cochrane Collaboration standard. We searched the literature database for randomized controlled trials (RCTs) published before January 2020. The databases that were searched included PubMed, EMBASE, Google, Web of Science, and Cochrane Library. The retrieval strategy was performed by the method of free words combined with the Medical Subject Headings (MeSH). The literature search was conducted using the keywords “Total Knee Arthroplasty,” “Kinematic Alignment,” “Kinematic,” “Mechanical Alignment,” “Mechanical,” and “biomarker” using Boolean operators (AND), (OR), and (NOT). Literature was retrieved without restricting the language.

### *Inclusion and Exclusion Criteria*

Criteria for inclusion: (i) randomized controlled trials; (ii) comparisons of clinical results between KA‐TKA and MA‐TKA in total knee arthroplasty; (iii) primary knee replacement surgery; (iv) observation indexes include “The Western Ontario and McMaster Universities (WOMAC) Osteoarthritis Index,”, “Knee Society Score (KSS),”“Oxford Knee Score (OKS),” “combined Knee Society Score (KSS),” “Knee injury and Osteoarthritis Outcome Score (KOOS),” ”EQ‐5D‐5L,” range of motion (ROM), lower limb alignment, ligament release, and complications; and v) studies published in English.

Criteria for exclusion: (i) basic research or cadaver study; and (ii) inaccessible data or full‐text.

### *Data Extraction*

Data from the studies from all selected articles were extracted independently by two of the authors using a data extraction template, which was designed before the database searches. From each study, first author, years, type of study and surgery, sample size, follow‐up time, and clinical outcome were extracted from the literature. Any disagreement was resolved through discussion and consensus or consultation with other authors in cases of disagreement. If the data from a study was missing, insufficient, or vague, we contacted the author or corresponding authors by email or telephone to retrieve further information.

### *Quality Evaluation*

Literature quality was evaluated independently by two of the authors with the Cochrane Collaboration Network risk evaluation tool. The risk of bias for each indicator was divided into three levels: “low,” “high,” and “unclear.” If we obtained more than 10 articles, a funnel chart or Eggers regression test was used to assess publication bias.

### *Observation Indexes*

#### *Western Ontario and* *McMaster* *Universities Osteoarthritis Index (WOMAC)*

The WOMAC is a validated questionnaire to evaluate lower extremity osteoarthritis and joint replacement. The WOMAC questionnaire produces three subscale scores (pain, stiffness, and physical function) and a total score. Patients are asked to answer each question about the severity of pain, stiffness, or behavioral difficulties experienced in the previous 48 hours. There are five response options ranging from “none” to “extreme” to choose. A response of “none” was scored as 0,”mild” as 1, “moderate” as 2, “severe” as 3, and “extreme” as 4. The scores of the questions in each subscale were summed together to get scores for pain, stiffness, and physical function. A lower subscale score indicates less pain, less stiffness, or better physical function. A total score of < 70 is considered a severe score, 21–48 is moderate, <21 is mild.

#### *Knee Society Score (KSS)*

The KSS is a condition‐specific validated questionnaire widely used to evaluate the functional capabilities of the knee joint before and after total knee arthroplasty. The scoring system consists of two parts. One part is the knee score. The assessment includes pain (maximum 50 points), stability (maximum 25 points), total range of flexion (maximum 25 points), and other items (varus, valgus, extension delay, and flexion contracture). The other part is the function score. The assessment includes walking distance (maximum 50 points), ability to climb stairs (maximum 50 points), and the use of walking aids. The highest score for each part is 100 points, and a higher score means better knee function. The evaluation result score is rated as four levels: 80–100 points, 70–79 points, 60–69 points, <60 points.

### *Statistical Analysis*

Statistical analysis of all extracted data was carried out using Review Manager 5.3 (The Cochrane Collaboration, Oxford, UK), with *P <* 0.05 considered statistically significant. Heterogeneity between studies was evaluated by calculating the *I* ^*2*^. If the *I* ^*2*^ was greater than 50%, it was considered high heterogeneity, and a random‐effects model was chosen to analyze the data; otherwise, the fixed‐effects model was applied. Enumerated data were presented as the risk ratio (*RR*) or odds ratio (*OR*) and 95%*CI*, while continuous data were presented as the weighted mean difference (*WMD*) or standardized mean difference (*SMD*) and 95%*CI* as a statistical measure of the curative effect. We attempted to use a funnel plot to evaluate the publication bias; a symmetrical funnel plot may indicate a low publication bias, while an asymmetric funnel plot may indicate possible publication bias.

## Results

### *Literature Search Results and Study Characteristics*

**Fig. 2:**
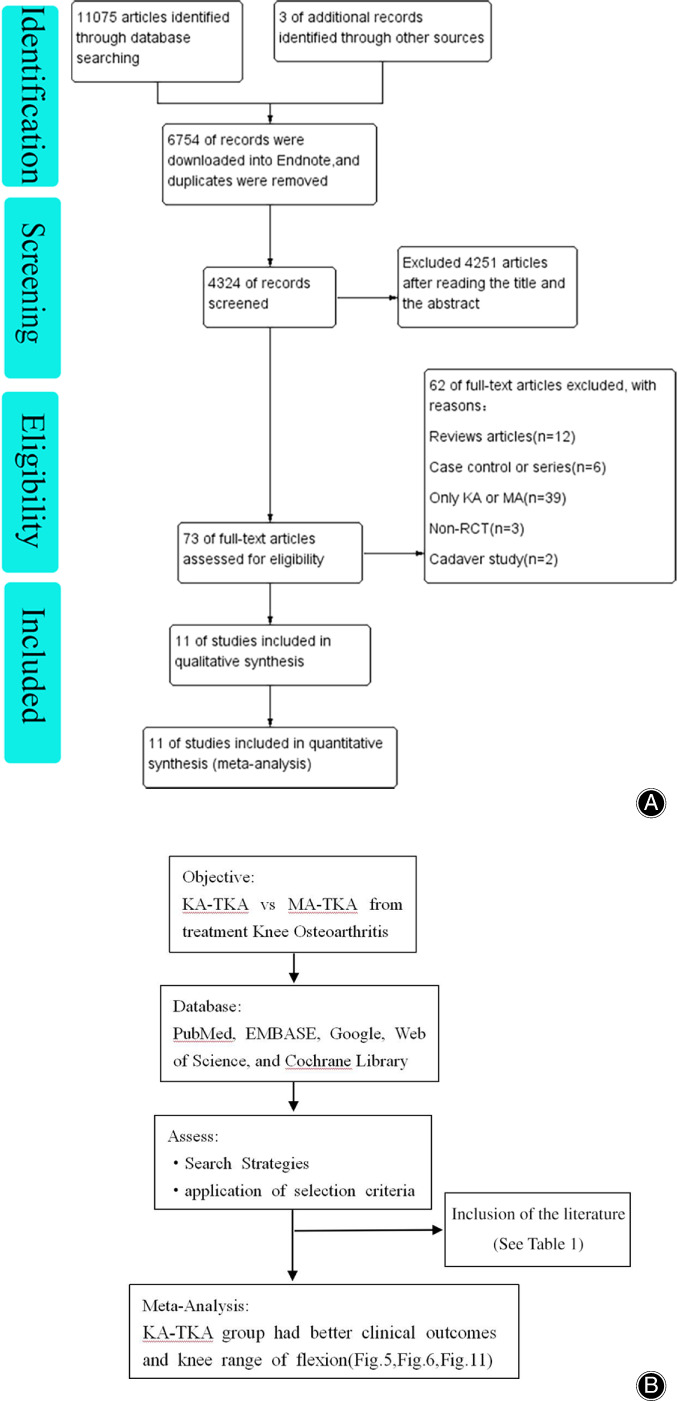
(A) Flow chart of literature processing. (B) The literature guide search and results.

We retrieved 11,075 papers from the databases; 6754 duplicates were removed by EndNote X9.1 software (Fig. 2). This left 4324 articles. Next, 4251 articles were excluded after reading the titles and abstracts, and the remaining 73 articles were retained for further evaluation by reading the full texts. Of these articles, 11 randomized controlled trials (RCTs)^13^, ^32^, ^33^, ^34^, ^35^, ^36^, ^37^, ^38^, ^39^, ^40^, ^41^ were included, with a total of 553 patients in the KA‐TKA group and 550 patients in the MA‐TKA group. The literature guide search and results are shown in Fig. 2B. The basic information of the 11 included articles is shown in Table 1.

**TABLE 1:** The basic information of the 11 RCT studies

Authors	Year	Study design	Total patients	Sample size (knees)		Follow‐up times (months)	Measurement index
				KA	MA		
MacDessi *et al*.^34^	2020	RCT	128	70	68	12	Operative time, FJS‐12, KOOS, HKA angle, LDFA, MPTA, KOOS, EQ‐5D‐5L
McEwen *et al*.^35^	2020	RCT	82	41	41	24	Tibial component slope angle, Femoral rotation angle, Ligament release,KOOS,OKS,FJS‐12,HKA angle, LDFA, MPTA, Extension/Flexion range
Yeo *et al*.^36^	2019	RCT	60	30	30	8.0 years	WOMAC, KSS, Flexion range
Laende *et al*.^37^	2019	RCT	47	24	23	24	Ligament release, UCLA, OKS, HKA angle, MPTA
Young *et al*.^38^	2017	RCT	99	49	50	24	Tibial component slope angle, Femoral rotation angle, Ligament release,WOMAC,KSS,OKS,FJS‐12,HKA angle,LDFA,MPTA,EQ‐5D‐5L,Flexion range
Calliess *et al*.^39^	2017	RCT	200	100	100	12	Tibial component slope angle, WOMAC, KSS, HKA angle, LDFA, MPTA
Waterson *et al*.^32^	2016	RCT	86	36	35	12	KSS,UCLA,KOOS,EQ‐5D‐5L,Flexion range
Dossett *et al*.^33^	2014	RCT	120	60	60	24	WOMAC,KSS,OKS,HKA angle,LDFA,MPTA,Extension/Flexion range
Matsumoto *et al*.^41^	2017	RCT	60	30	30	12	KSS,Extension/Flexion range
Dossett *et al*.^13^	2012	RCT	120	41	41	6	Operative time, WOMAC, KSS, OKS, HKA angle, Extension/Flexion range
Claudio *et al*.^40^	2015	RCT	144	72	72	6	KSS

FJS‐12, Forgotten Joint Score‐12; LDFA, Lateral distal femoral angle; MPTA, Medial proximal tibial angle; UCLA, University of California, Los Angeles Activity Score.

### *Risk‐of‐bias and Publication Bias Assessment*

**Fig 3:**
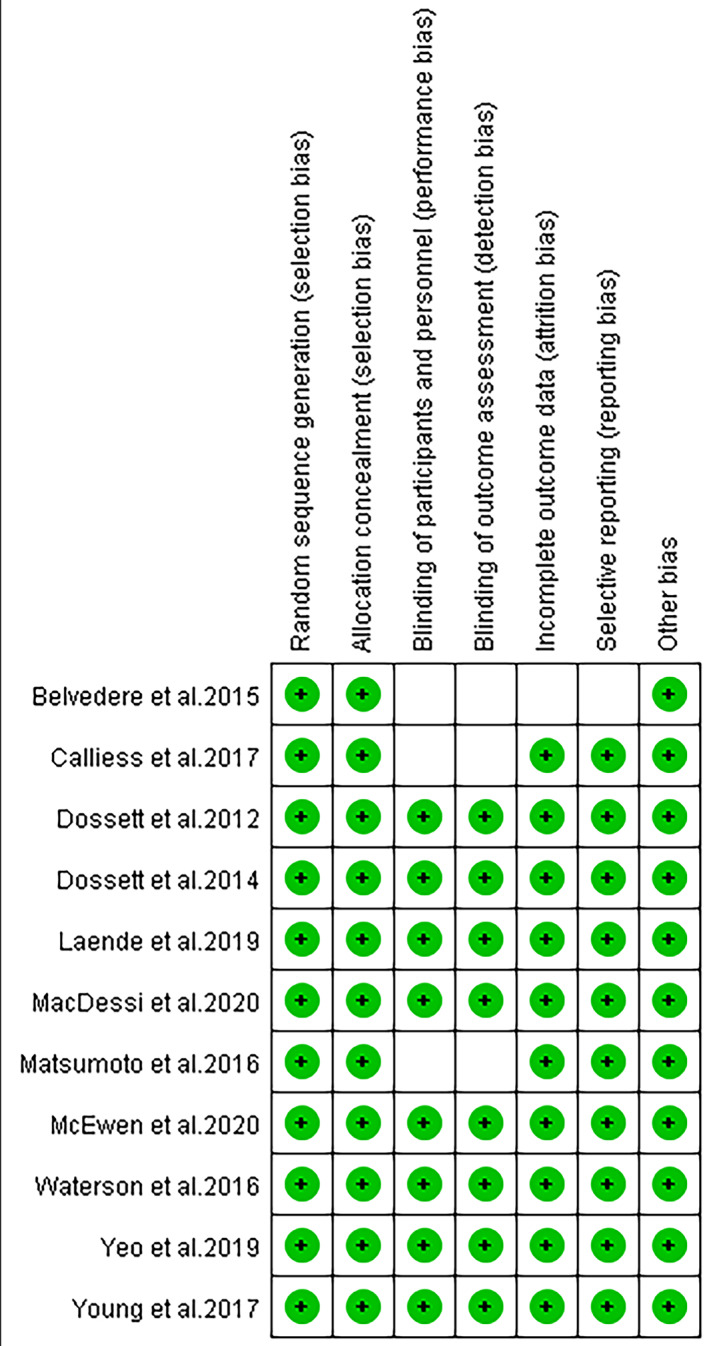
Eleven articles underwent Risk‐of‐Bias Assessment summary.

**Fig. 4:**
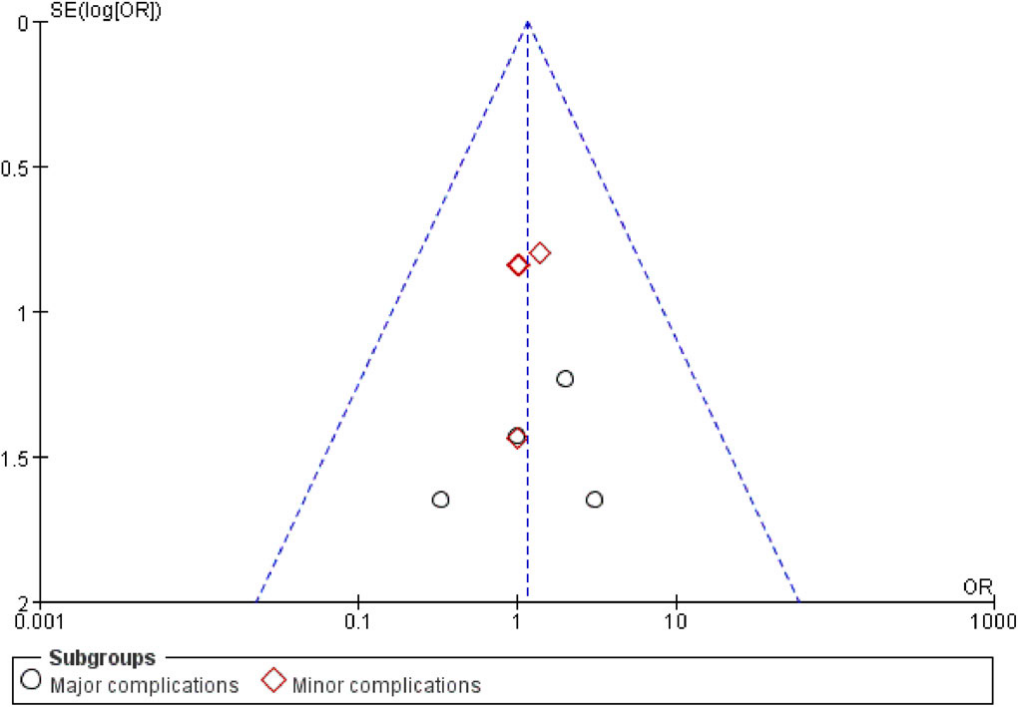
The funnel plot for the symmetrical may indicate a low publication bias.

The 11 papers included were evaluated for risk of bias according to the seven aspects in Fig. 3, which shows that all the RCT articles had a low risk of bias. The symmetrical funnel plot may indicate low publication bias (Fig. 4).

### *Clinical Outcomes*

**Fig. 5:**
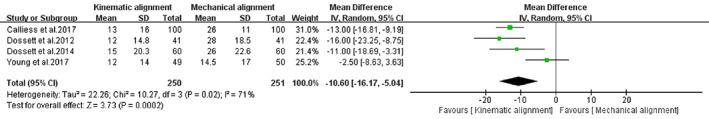
The forest plot for WOMAC (0–96 best–worst).

Five randomized controlled trials^13^, ^33^, ^36^, ^38^, ^39^ with a total of 599 patients evaluated WOMAC scores. We detected high heterogeneity between the KA‐TKA and MA‐TKA groups (*I* ^*2*^ *=* 93%). We found that one of these studies^36^ reported the results in the long term (8‐year follow‐up), while other studies reported a short‐term follow‐up. We excluded this article from our meta‐analysis for further analysis. The meta‐analysis result was heterogeneous (*I* ^*2*^ *=* 71%), so the random‐effects model was used for further analysis. The results showed that the WOMAC score of the MA‐TKA group was higher than that of the KA‐TKA group [*MD* = ‐10.60, 95%*CI* (−16.17, −5.04), *P* = 0.0002, Fig. 5].

**Fig. 6:**
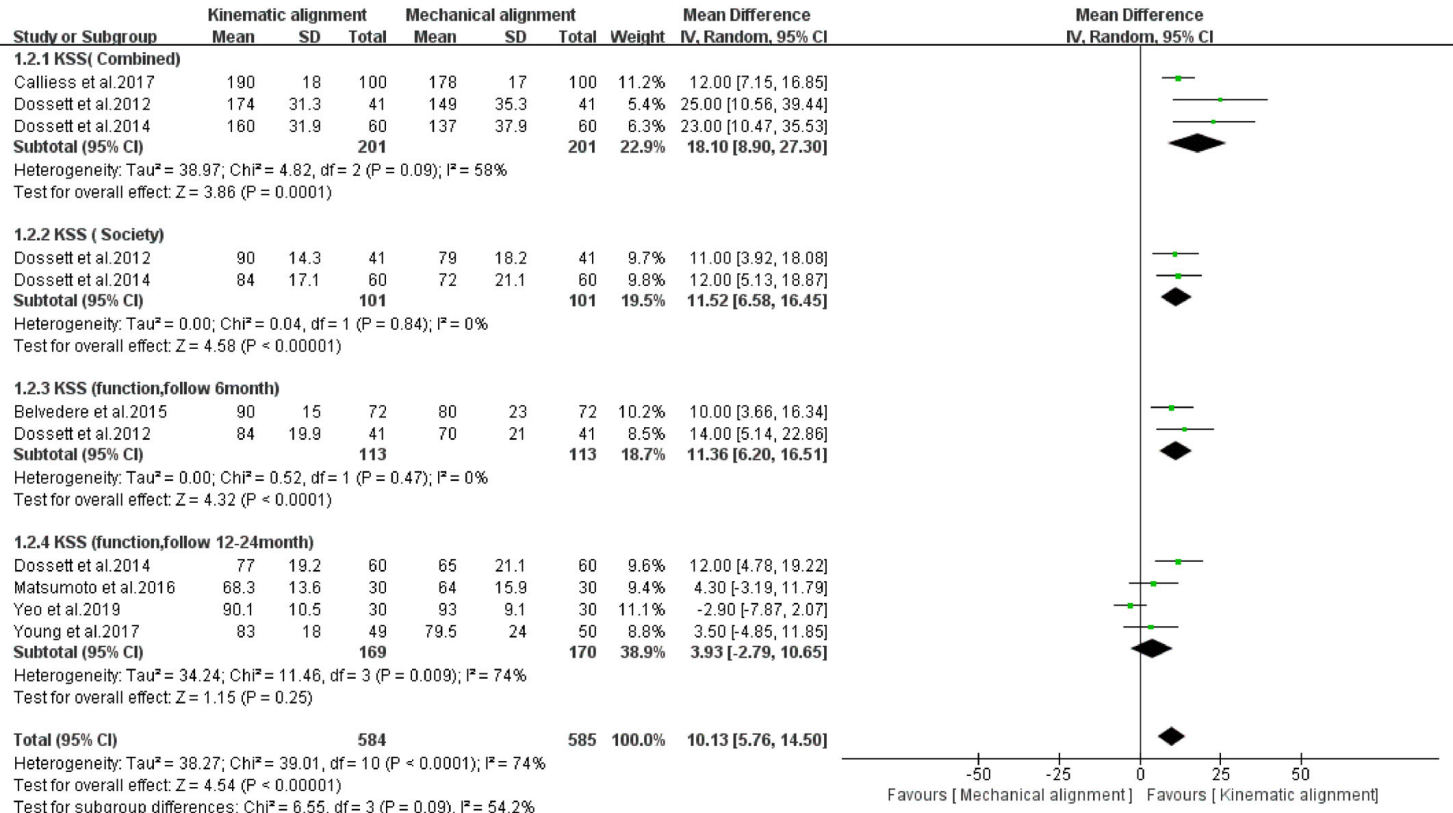
The forest plot for Combined Knee Society score (KSS, 0–200 worst–best), Knee Society Score(0–100 worst–best), Knee function Score(0–100 worst–best).

Knee joint function and pain scores were evaluated by the KSS and OKS. The total knee scores of the KA‐TKA group were better than those of the MA‐TKA group, and the differences were statistically significant [*I* ^*2*^ *=* 74%, *MD* = 10.13, 95%*CI* (5.76, 14.50), *P* < 0.00001, Fig. 6]. Three trials with a total of 402 patients evaluated the combined KSS. The random‐effects model was used instead of a fixed‐effects model due to the high heterogeneity (*I* ^*2*^ *=* 84%) of the combined KSS. The combined KSSs were better in the KA‐TKA group than in the MA‐TKA group, and the difference between the groups reached statistical significance [*MD* = 18.10, 95%*CI* (8.90, 27.30), *P* = 0.0001, Fig. 6].

**Fig. 7:**
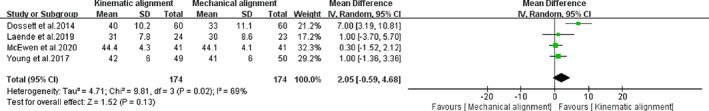
The forest plot for Oxford Knee Score(0–48 worst–best).

Two trials^36^, ^38^ with a total of 238 patients evaluated the KSS. The results indicated that the mean scores of the KA‐TKA group were higher than those of the MA‐TKA group [MD = 11.52, 95%*CI* (6.58, 16.45), *P* < 0.00001, Fig. 6]. Six trials^13^, ^33^, ^36^, ^38^, ^40^ with a total of 565 patients evaluated the knee function score. According to the different scoring times, 6 months^13^, ^40^ and 12–24 months,^33^, ^36^, ^38^ the included studies were divided into two subgroup analyses. Two trials^13^, ^40^ followed up 226 enrolled patients for 6 months, and based on our findings, the KA‐TKA group had higher mean scores than the MA‐TKA group [*MD* = 11.36, 95%*CI* (6.20, 16.51), *P* < 0.0001, Fig. 6]. Four trials^33^, ^36^, ^38^ followed up 339 enrolled patients for 12–24 months, and the results showed that the two groups had similar mean scores [*MD* = 3.93, 95%*CI* (−2.79, 110.65), *P =* 0.25, Fig. 6]. Five trials^13^, ^33^, ^35^, ^37^, ^38^ with a total of 430 patients evaluated OKS. However, one study^13^ calculated the scores differently from the others, and hence, we excluded this article from this study. The meta‐analysis result was heterogeneous (*I* ^*2*^ *=* 69%), so the random‐effects model was used for further analysis. The results showed that the two groups had similar mean scores [*MD* = 2.05, 95%*CI* (−0.59, 4.68), *P =* 0.13, Fig. 7].

**Fig. 8:**
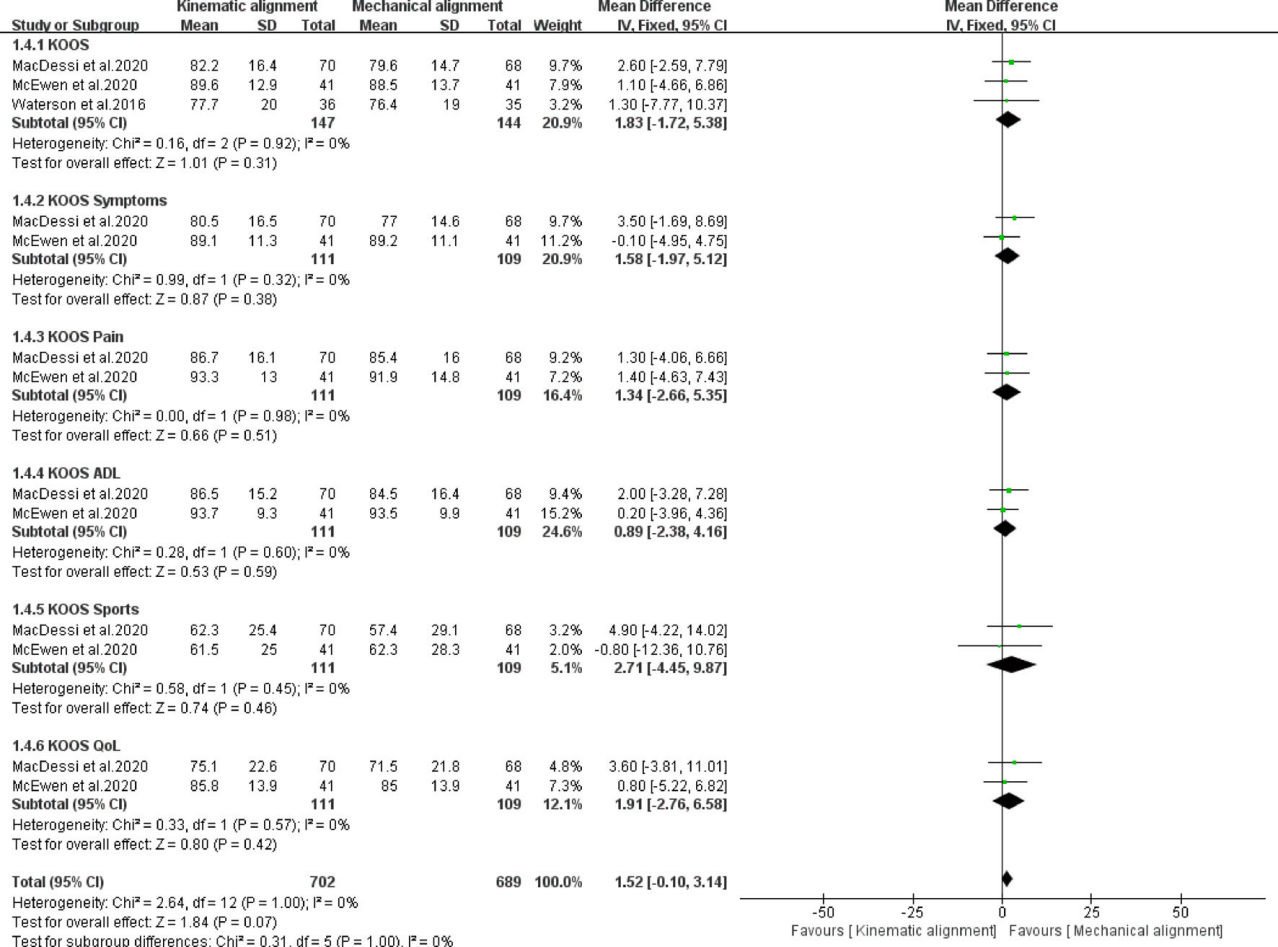
The forest plot for knee injury and osteoarthritis score (KOOS, 0‐100 worst–best).

**Fig. 9:**
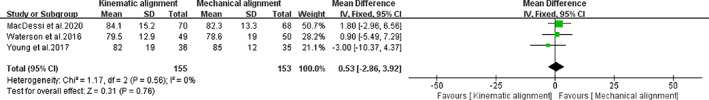
The forest plot for EQ‐5D‐5L.

Quality of life (QoL) was evaluated using KOOS and EQ‐5D‐5L. Three trials with a total of 291 patients evaluated KOOS. The meta‐analysis result showed low heterogeneity (*I* ^*2*^ *=* 0%), and hence, a fixed‐effects model was used for further analysis. Figure 8 shows that these two groups had similar mean scores in terms of KOOS [*MD* = 1.83, 95%*CI* (−1.72, 5.38), *P =* 0.31], KOOS symptoms [*MD* = 1.58, 95%*CI* (−1.97, 5.12), *P =* 0.38], KOOS pain [*MD* = 1.34, 95%*CI* (−2.66, 5.35), *P =* 0.51], KOOS ADL [*MD* = 0.89, 95%*CI* (−2.38, 4.16), *P =* 0.59], KOOS sports [*MD* = 2.71, 95%*CI* (−4.45, 9.87), *P =* 0.46], and KOOS QoL [*MD* = 1.83, 95%*CI* (−2.76, 56.58), *P =* 0.42]. Three trials with a total of 291 patients evaluated EQ‐5D‐5L. The meta‐analysis result showed low heterogeneity (*I* ^*2*^ = 0%), and hence, a fixed‐effects model was used for further analysis. The results showed that the two groups had similar mean scores [*MD* = 0.53, 95%*CI* (−2.86, 3.92), *P =* 0.76, Fig. 9].

### *Lower Limb Alignment*

**Fig. 10:**
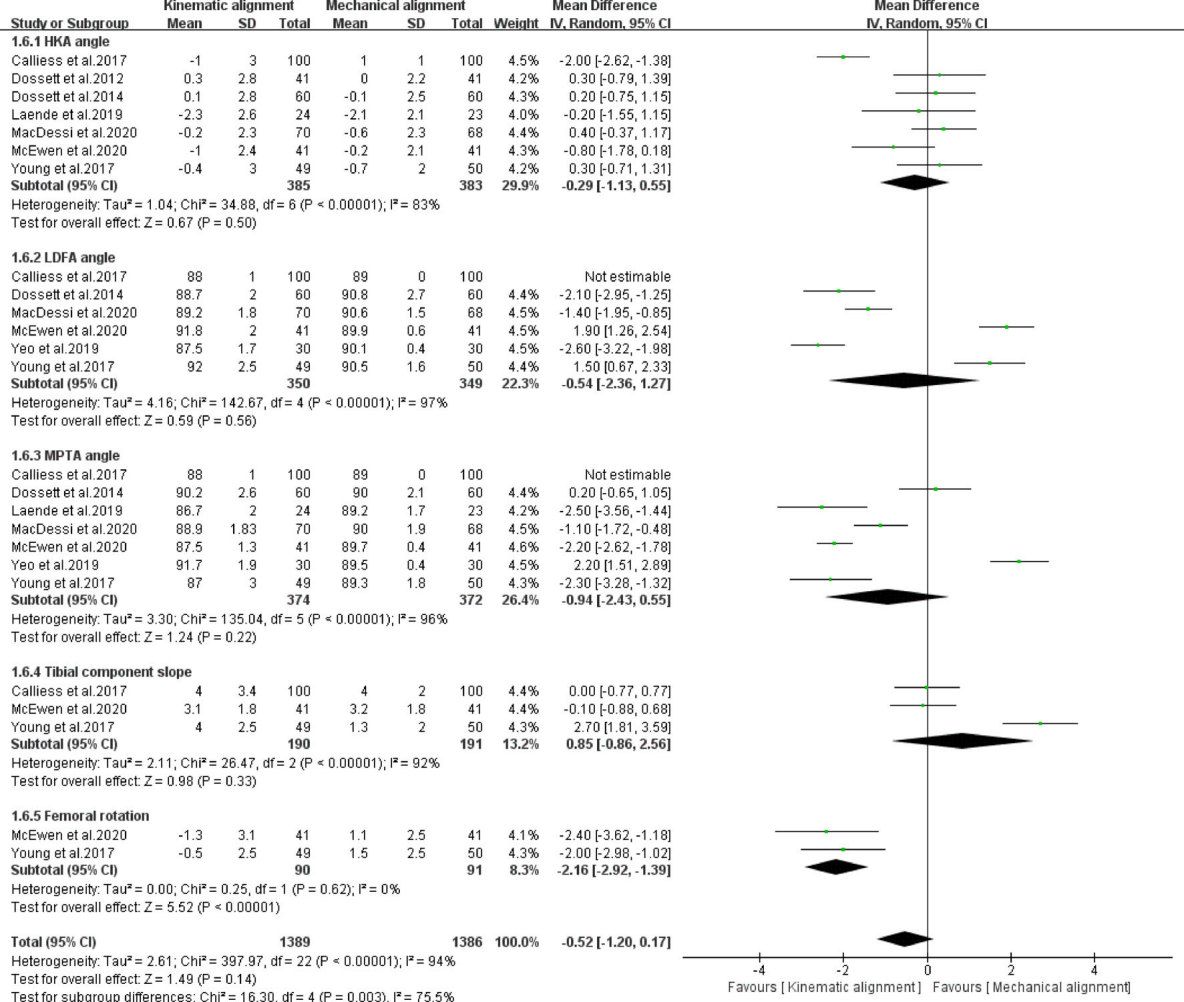
The forest plot for HKA, LDFA, MPTA, tibial component slope and femoral rotation angle.

Basic lower limb alignment measurements should include the HKA angle, tibial component slope angle, femoral component rotation to sulcus line angle, LDFA, and MPTA angle. The random‐effects model was used instead of a fixed‐effects model since there was heterogeneity. Figure 10 shows that the two groups had similar mean scores in terms of HKA angle [*I* ^*2*^ = 83%, *MD* = ‐0.29, 95%*CI* (−1.13, 0.55), *P =* 0.50], tibial component slope angle [*I* ^*2*^ = 92%, *MD* = 0.85, 95%*CI* (−0.86, 2.56), *P =* 0.22], LDFA angle [*I* ^*2*^ = 97%, *MD* = ‐0.54, 95%*CI* (−2.36, 1.27), *P =* 0.38], and MPTA angle [*I* ^*2*^ = 96%, *MD* = 0.94, 95%*CI* (−2.43, 0.55), *P =* 0.22]. The results of two trials with 181 patients showed that the femoral component internal rotation to sulcus line angles of the two groups were different [*I* ^*2*^ *=* 0%, *MD* = ‐2.16, 95%*CI* (−2.92, −1.39), *P<*0.00001, Fig. 10]. From the data in Fig. 8, the rotation angle of the KA‐TKA group still had a varus alignment, whereas the MA‐TKA group presented with a valgus pattern.

### *ROM*

**Fig. 11:**
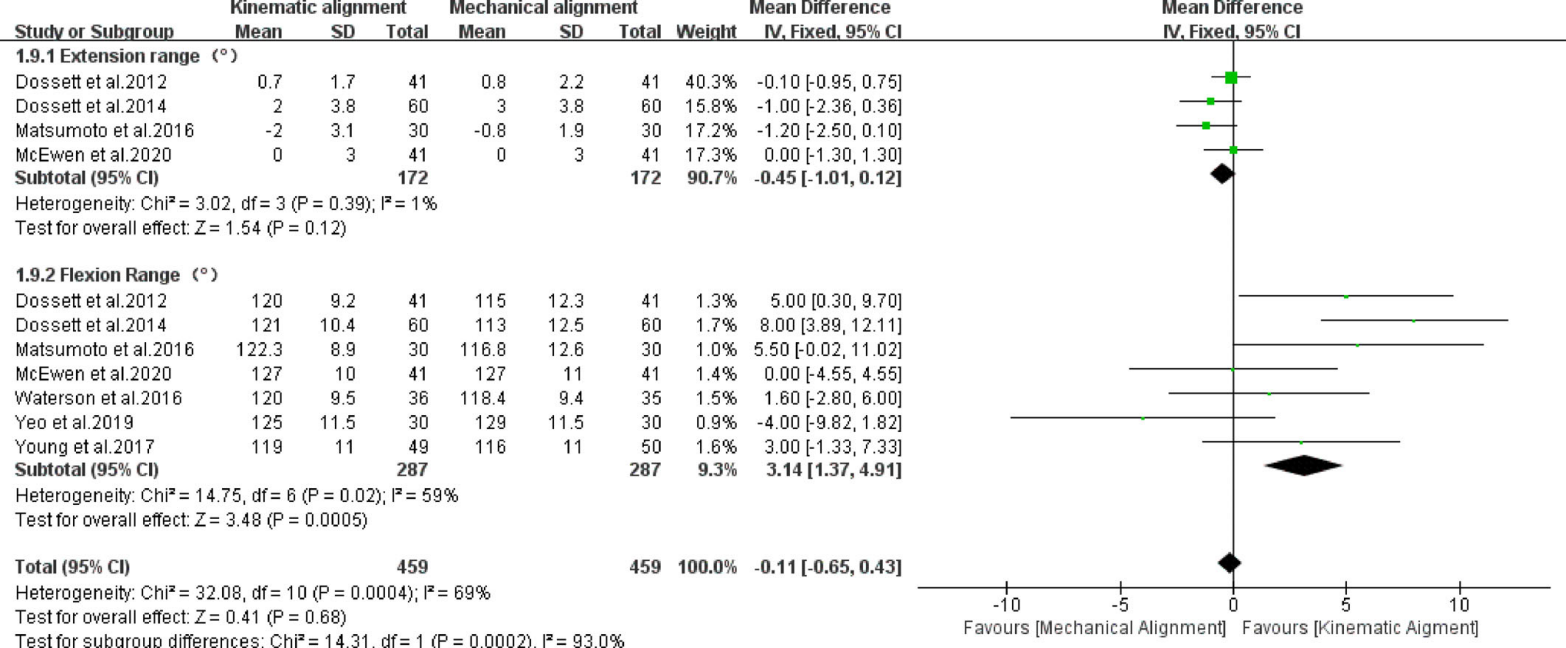
The forest plot for extension/flexion range of knee.

Our review found a total of seven relevant studies, including four studies^13^, ^33^, ^35^ involving extension range angle and seven studies involving flexion range angle^13^, ^32^, ^33^, ^35^, ^36^, ^38^. As seen from Fig. 11, the difference in extension range angle did not reach statistical significance [*I* ^*2*^ = 1%, *MD* = ‐0.45, 95%*CI* (−1.01, 0.12), *P =* 0.12] but the difference in flexion range angle did [*I* ^*2*^ = 59%, *MD* = 3.14, 95%*CI* (1.37, 4.91), *P =* 0.0005]. Furthermore, the KA‐TKA group had a higher mean flexion range angle than the MA‐TKA group (*P =* 0.0005).

### *Ligament Release and Complications*

**Fig. 12:**
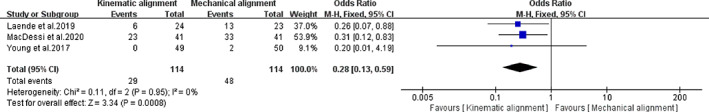
The forest plot for ligament release.

Three articles^35^, ^37^, ^38^ with 228 enrolled patients reported ligament release. Intraoperatively, 77 cases of ligament releases were recorded, with 29 cases in the KA‐TKA group and 48 cases in the MA‐TKA group. The fixed‐effects model was used instead of a random‐effects model due to the low heterogeneity (*I* ^*2*^ = 0%). The incidence of ligament release was lower in the KA‐TKA group than in the MA‐TKA group [*MD* = 0.28, 95%*CI* (0.13, 0.59), *P =* 0.0008, Fig. 12].

**Fig. 13:**
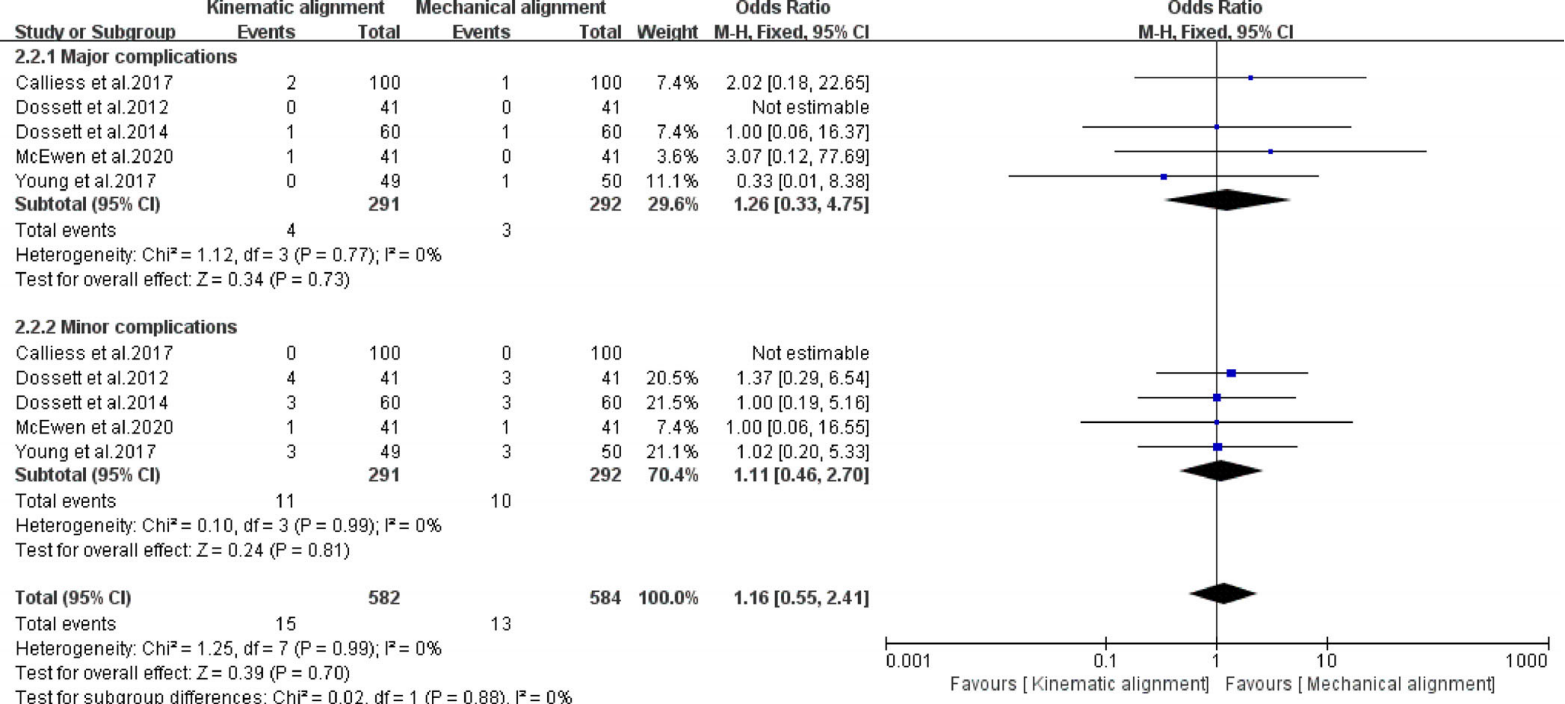
The forest plot for complication. Major complications are defined as revision of knee joint or removal of prosthesis caused by various reasons; and other additional surgery treatments were classified as minor complications.

Of the 11 studies, five studies^13^, ^33^, ^35^, ^38^, ^39^ provided data regarding complications (KA‐TKA: 15/582; MA‐TKA: 13/584). There was no statistical significance between the two groups (*I* ^*2*^ = 0%, *MD* = 1.16, 95%*CI* (0.55, 2.41), *P =* 0.70, Fig. 13). All the complications were clustered into two subgroups: subgroup 1 (major complications) and subgroup 2 (minor complications). Figure 13 shows that these two groups had the same result in terms of major complications [*I* ^*2*^ = 0%, *MD* = 1.26, 95%*CI* (0.33,4.75), *P =* 0.73] and minor complications [*I* ^*2*^ = 0%, *MD* = 1.11, 95%*CI* (0.46,2.56), *P =* 2.70].

## Discussion

### *Results on the* *Meta‐Analysis*

The 11 studies (RCTs) that fulfilled the inclusion criteria included 1103 participants: 553 patients in the KA‐TKA group and 550 patients in the MA‐TKA group. Furthermore, follow‐up ranged from 6 months–8 years. Most of the literature reported that the patients were followed up for 6–24 months. The results of the KA‐TKA group were better than those of the MA‐TKA group in terms of the WOMAC score, combined KSS, KSS, knee function score, and knee range of flexion, while the EQ‐5D‐5L, KOOS, KOOS (symptoms, pain, ADL, sports, and QoL), complications, knee range of extension, HKA angle, tibial component slope angle, LDFA, and MPTA angle in the KA‐TKA group were not significantly different from those in the MA‐TKA group. The incidence of ligament release in the KA‐TKA (29/144) group was lower than that in the MA‐TKA (48/144) group, and the difference was statistically significant.

### *Functional Outcome After* *KA‐TKA*

Although the survival rate and the clinical and functional outcomes of TKA are very good overall, approximately 20%–25% of patients remain unsatisfied with the outcome^8^. There are undoubtedly many reasons, but the two main reasons are as follows. (i) Reports have indicated that 98% of normal limb femoral and tibial mechanical axes are not in a straight line and that 76% of normal limbs exceed the range of 3° of the neutral mechanical axis^16^. Bellemans *et al*.^42^ studied 250 young adults without arthritis and showed that the rate of constitutional varus knees was 24.6%, with a rate of 32.0% for males and 17.0% for females. If these patients need to be treated by MA‐TKA, the clinical outcomes of patients will still be poor after surgery. (ii) MA‐TKA does not entirely restore knee joint kinematics, kinetic characteristics can irritate the soft tissue, and imbalance of the knee joint has attracted the full attention of domestic and foreign scholars. The proposed kinematic alignment is based on the concept of the kinematic axes of the knee and their relationship to the femoral condyles^14^, ^15^. KA‐TKA does not restore the HKA angle of the limb to neutral, it mainly considers the three‐dimensional alignment of the components relative to the knee, which may lower the frequency of ligament release and improve clinical effectiveness^19^, ^36^, ^37^, ^38^. Recently, two RCTs compared KA *vs* MA in TKA, and KA‐TKA reduced the incidence of ligament release^35^, ^37^. Our meta‐analysis revealed that the rate of ligament release in MA‐TKA was higher than that in KA‐TKA (*P =* 0.0008). Consistent with findings in previous studies^27^, ^31^, ^33^, ^36^, ^38^, ^39^, ^40^, we found that the KA‐TKA group had better knee function outcomes than the MA‐TKA group in terms of the WOMAC score, KSS, combined KSS, and KSS.

### *Complications and Survival*

Although the goal of KA‐TKA is to restore normal knee kinematics or prearthritic kinematics and restore the patient to previous functional levels, the concern for increased risk of patellofemoral instability and polyethylene wear was raised^29^, ^30^, ^43^, ^44^, ^45^. Ishikawa *et al*.^46^ found that patients with more significant femoral rollback and external rotation of the femur can obtain better restoration of the motion of tibia flexion‐extension after KA‐TKA. Our updated meta‐analysis found that the KA‐TKA group had a higher mean flexion range angle than the MA‐TKA group (*P =* 0.0005). However, Ishikawa *et al*.^46^ expressed concern about varus alignment of the tibial prosthesis, which can increase the risk of polyethylene wear, leading to reduced prosthesis survival, and which also increases contact stress at the patellofemoral joint and may cause patellofemoral joint instability. Our meta‐analysis reported three patients with patellar instability: two patients in the KA‐TKA group^13^, ^38^ and one patient in the MA‐TKA group^38^. The results showed that the two groups had similar rates of complications (KA‐TKA: 15/582, MA‐TKA: 13/584, *P =* 0.70). Howell *et al*.^24^ prospectively followed 214 knees subjected to KA‐TKA, and the mean follow‐up was 38 months (31–43 months). There has been great interest in investigating varus (>3°) or valgus (<‐3°) knee alignment, and there was no polyethylene wear or loosening leading to loosening of the revision prosthesis in comparison with alignment of the HKA angle in range (0° ±3°). Another study showed that after patients underwent the KA‐TKA, 80% presented with varus alignment of the tibial component, and 70% had a varus alignment of the limb. However, varus alignment of the tibial component and limb did not adversely affect implant survival or function during the follow‐up periods. Of the patients whose follow‐up was 3 to 8 years (mean, 6.3 years), only two cases of loosening of the prosthesis were considered failures; the survival rate was 97.5% and the revision rate was 0.4%^47^. Howell *et al*.^48^ show that the overall survival rate of prostheses hold on pleasurable，the 10‐years survival rate of approximately 97.5%. Yeo *et al*.^36^ reported the follow‐up of patients after KA‐TKA and MA‐TKA for 8 years, and they obtained similar clinical and radiological results. Thus, they suggested that the increased risk of surgical failure after KA‐TKA may not hold. However, KA‐TKA as a new option for the treatment of patients with KOA lacks long‐term results, so it is necessary to collect detailed observations, perform studies with longer follow‐up and data analysis, and scientifically evaluate the new treatment methods.

### *Limitations*

However, some limitations still existed in this study. First, high heterogeneity existed in some comparisons. Although we used several subgroups, heterogeneity was still present in some results, and we failed to thoroughly explain the heterogeneity. Second, although the short‐term clinical effect after KA‐TKA has been evaluated, there is a lack of long‐term follow‐up results for the survival rate of the prosthesis. Finally, the chief limitation of this study was the small sample size; thus, a large sampler size, more rigorous RCTs, and longer follow‐up supporting these results will be needed in the future.

### *Conclusion*

This meta‐analysis shows that the KA‐TKA had better outcomes than the MA‐TKA on WOMAC score, Combined KSS, KSS (Society and Function) score, and knee range of flexion at short‐term follow‐up.

## Disclosure

The authors declare that we have no conflict of interest.
